# Supplementary Courses of Treatment Required for Chronic Nasal Inflammation

**Published:** 1896-10

**Authors:** Thos. F. Rumbold

**Affiliations:** St. Louis, Mo.


### SUPPLEMENTARY COURSES OF TREATMENT REQUIRED FOR CHRONIC NASAL INFLAMMATION.

BY THOS. F. RUMBOLD, M.D.,
ST. LOUIS, MO.

By supplementary courses of treatment for chronic inflammation of the nasal passages is meant a renewal of the local applications at such seasons of the year as the patient is most liable to have renewed colds in his nasal passages, throat, and ears. They are given to a patient who has been under the care of a physician long enough—from two to five months, if an adult—to reduce his nasal inflammation to such a condition that he does not complain of any of his former symptoms, but not long enough for the inflamed membrane to have completely recovered. In such a case the secretion from the mucous surfaces has assumed nearly its normal character, and quantity, and all abnormal growths, should there have been any, have been removed from the nose, throat, and ears.

*Importance of Supplementary Treatment.*—During my pupilage with Dr. S. D. Gross, in Philadelphia, in the fall of 1861, I was fortunate enough to pay attention to the remarks of some intelligent patients concerning the value of a few treatments given at the early portions of the spring or the fall. At the time I thought that some patients took cold more easily in the spring, and others more easily in the fall. In either case a prophylactic course was recommended. I was more fully impressed with the benefit of this method while treating a number of cases of nasal disease in the United States General Hospital, at Jefferson Barracks, Mo., from 1862 to 1865. While in this hospital I had a number of such patients under my daily sight for nearly three years, so that I was well able to judge of the liability of the disease to return at the changes of the seasons, even under the most favorable circumstances. While in this hospital, I found that instead of giving a few treatments, either in spring or in fall, that is, in whichever season they took cold more easily and severely, they should be treated both spring and fall, that is, two seasons after the first course, instead of one.

In a paper I read in the St. Louis Medical Society in June, 1896, on the "Treatment of Simple and Ulcerative Rhinitis," I mentioned the importance of such treatments. The points of the paper were the hygienic rules, which I directed my patients to follow during, and after treatment, and "the necessity for spring and fall treatments to maintain the still weakened mucous membrane in such a condition that it could in time recover its normal defensive power, so as to withstand the inclemency of the weather at the changes of the seasons of the year"; both points absolutely essential to the ultimate cure of this disease. By this time I found that more than two seasons of supplementary treatments were required for most patients.

This paper was published in pamphlet form in 1870. In my work on the "Treatment of Nasal Catarrh," 1881, on page 255, I stated: "These fall and spring treatments (for patients from twenty to forty years of age) will have to be repeated from three to five years; and for the oldest class, they will have to be repeated fall and spring, or, fall or spring maybe during their lifetime"; showing that I had again increased the number of supplementary treatments. In my work on the "Medical and Surgical Treatment of Catarrhal Diseases of the Nose, Throat, and Ears," 1886, p. 476; and again, in the *Journal of the American Medical Association*, June, 1893, page 885, I repeated the same thing.

*The Liability of the Return of the Disease.*—One of the reasons for the general impression that nasal inflammation is incurable—an impression that exists among the medical profession and is held by the laity—is that the disease is universally observed to give evidence of return at the next change of the season, after the primary long course of the treatment, either spring or fall. This return is observed in all patients, from the tenth, or even, in some instances, from the fifth year of age, upward. The return is taken as an evidence that the first or primary long, expensive course of treatment did not cure the case, and be it marked, this is true; but if a few treatments with the spray producer are given at the right time, it will be found that all of these symptoms disappear at once, at least, in a very much shorter time than during the primary course. Generally the supplementary treatments in the spring and fall amount to about ten per cent. of the number of the primary course. For example, if a patient has required fifty or seventy treatments during the primary course, the supplementary treatments will be about five or seven during the spring and fall. If the patient takes good care of himself, the number of these spring and fall treatments will gradually decrease year by year, as his mucous membrane returns to its normal condition, and it always does return to this condition if the patient—under forty-five years of age—takes good care of himself. As all the growths have been removed during the primary course, the case is one of uncomplicated nasal inflammation, and this inflammation is not of such an atonic character, and disappears under a few treatments.

The ultimate curability of this disease is a frequent subject of discussion during the primary course of treatment of a patient. After he has been under care for a few days, long enough for me to be enabled to judge as to the length of time required for this primary course, I tell him, as agreed upon the day of the first visit, about the number of treatments that will then be required, and about the number of courses of supplementary treatments, as well as the number of treatments of each of these courses. The whole subject is usually talked over, so that there is a perfect understanding, and with this understanding the patient places himself under my care for a term of years, the length of which will be according to the chronicity and severity of the disease.

Almost always the patient will voluntarily say that he will not forget to call for his spring and fall treatment; but should he do so, I write him a letter, again going over what I said to him during the preliminary course of treatment, plainly telling him that the supplementary treatments are required to continue the healing process commenced by the treatments he has received. The fact that the patient has been relieved of troublesome subjective and objective symptoms renders him unconscious of the necessity of further treatment, and the dread of further increasing his expenses is an additional reason for keeping away from his physician, notwithstanding the promise to return; but the certainty of the return of the disease and the uncertainty of the return of the patient make it imperative that he be reminded by letter concerning the importance of the supplementary courses.

While it is a matter of importance to the rhinologist's reputation that these supplementary treatments should be given, it is sometimes also a matter of great difficulty to get the patient to return for them, and were it not that a promise of a cure or of an alleviation, as the case may be, has been made upon these conditions, it would be very convenient to let the patient go without a written notification. If he does not come, consequently is not cured, he adds additional facts to the report of the incurability of the disease, and says that his physician did not perform his promise. This alone is what makes it worth while to keep him in sight, and under treatment. In five to seven years the number of these uncured, delinquent patients will be so great and their complaints so continuous, that they will have a very injurious effect on one's practice. It is plainly seen that the only way to prevent this injury is to have them return for the supplementary courses. In a word, they must be cured, and the supplementary courses are essential to their complete recovery.

Toward the end of these supplementary courses, the patient's mucous membrane may be so nearly in a normal condition that ordinary exposures will have no ill effect upon this once excessively sensitive membrane; therefore, he may miss a spring or a fall course, or he may pass a year or so, and then require but one or two local applications.

Too much emphasis cannot be laid upon the importance of curing curable cases by the supplementary treatments, and of continuing the spring and fall treatment with those whose age or the severity of the disease precludes a cure, cases in which a promise of relief only has been given. The measure of success following my practice in the treatment of this disease, in a large proportion of my patients, justifies me in this course.

The best seasons of the year are spring and fall; usually from March 15th to May 15th, and from September 15th to November 15th. These dates are in the seasons of the greatest change or variance of temperature and moisture, and I have found, from many years' careful observations, more persons require treatment at these seasons than during any other periods of the year.

*The Number of Supplementary Courses.*—The age of the patient, which nearly always agrees with the grade of the inflammation, will do much to determine the number of the supplementary treatments at each course, and the number of years that these courses should be repeated, also the number and extent of the growths removed will have a determining influence as to the number of supplementary treatments. A boy of fifteen, who has required the removal of a large number of growths from his upper air passages is in need of a greater number of these courses than a person of even thirty years of age, who has not required the removal of abnormal growths.

The infant may require two to four treatments at the next change of the season after its first or primary course. If it has secretion flowing from the nasal passages, or if it has acquired growths on the tonsils, then it certainly will require a supplementary course. The mucous membrane, even at this age will not have completely recovered during the primary course of treatment. These treatments are given at proper intervals during the sixty days that form the season for supplementary treatment.

Children from the third to the tenth year of age, even with uncomplicated nasal inflammation may require a few supplementary treatments, usually from four to six of these treatments for one or two seasons; while those who have had growths removed or have had ear complications, will require these treatments for two years, at least.

Youths from ten to twenty years of age, having uncomplicated nasal inflammation will require from five to ten treatments during each supplementary course for two years. A case complicated with growths or with deafness will require two more of these courses. If these supplementary courses are not taken the disease will certainly return, as will the growths, and in a few years the patient will be in as bad a condition as though no treatments had been given, or no growths had been removed. This must be plainly and repeatedly told the patient.

Adults almost universally have nasal inflammation of so severe a character and of so long continuance, that they have a large number of nasal growths and many also have growths in the throat, and sometimes in the ears, as well as lung or other complications. They will require from eight to fifteen treatments at each change of the season for from three to five years.

It should not be expected that the blood-vessels of the mucous membrane and of the sub-mucous tissues, which have been enlarged to twenty or forty times their normal diameter, would be reduced to their normal diameters by the curative effect of a few months' local application. Such an expectation could be expressed only by a person totally unacquainted with the pathology of naso-mucositis. Even considering the mucous membrane alone, it is still in an atonic condition; and although the inflammation is greatly reduced, and all the former painful symptoms absent, yet the membrane must be maintained in an unirritated state for from three to five years before complete recovery can take place, before nature can make complete reparation. It requires this length of time, and the correction and protection of the supplementary courses to allow it time to regain its normal resisting power, and it will not have done this until its blood-vessels and nerves have returned to their normal condition. As soon as this has taken place, the patient, instead of taking cold more frequently than any one around him, will be no more liable to take cold in his nasal passages, throat, and ears than in other organs of his body. This is a positive evidence of his complete recovery; after this no more supplementary courses are required. In case these courses are not taken, the inflammation will certainly commence to increase with the first colds, which are usually taken at the change of the seasons.

If the patient is forty-five years of age, or older, the return of growths very seldom takes place; even if the inflammation returns with unusual severity, the extension of the disease is toward other organs, notably the eyes, the lungs, the stomach, and the brain. If the first, it is shown by the rapid changes of the anomalies of the vision; if the last, it is shown by unusual acts of forgetfulness, unusual irritability of temper, and by symptoms that are frequently called nervous prostration, etc. In a very few instances insanity results from the extension of this disease to the brain.

